# MBD2 and MBD3: elusive functions and mechanisms

**DOI:** 10.3389/fgene.2014.00428

**Published:** 2014-12-09

**Authors:** Roberta Menafra, Hendrik G. Stunnenberg

**Affiliations:** Department of Molecular Biology, Radboud UniversityNijmegen, Netherlands

**Keywords:** DNA methylation, methyl-CpG binding domain proteins, MBD2, MBD3, transcription regulation, chromatin immunoprecipitation

## Abstract

Deoxyribonucleic acid methylation is a long known epigenetic mark involved in many biological processes and the ‘readers’ of this mark belong to several distinct protein families that ‘read’ and ‘translate’ the methylation mark into a function. Methyl-CpG binding domain proteins belong to one of these families that are associated with transcriptional activation/repression, regulation of chromatin structure, pluripotency, development, and differentiation. Discovered decades ago, the systematic determination of the genomic binding sites of these readers and their epigenome make-up at a genome-wide level revealed the tip of the functional iceberg. This review focuses on two members of the methyl binding proteins, namely MBD2 and MBD3 that reside in very similar complexes, yet appear to have very different biological roles. We provide a comprehensive comparison of their genome-wide binding features and emerging roles in gene regulation.

## INTRODUCTION

### DNA METHYLATION

DNA methylation of cytosine residues was reported to be involved in gene silencing as early as 1975 (Holliday and Pugh, 1975; Holliday, 1989) and represents the first epigenetic mark (Holliday, 1989). In mammals, the predominant form is cytosine methylation (5mC) found within the context of paired symmetrical methylation at CpG dinucleotides. In mammalian genomes, around 70% of CpG dinucleotides are methylated (Robertson and Jones, 2000), however, CpG islands (CGIs) – regions of local high CpG density – are mainly unmethylated. CGIs constitute around 60% of human promoters (Bird, 1986). Methylation of CGI promoters results in transcriptional silencing, for example during genomic imprinting and X-chromosome inactivation (Feinberg and Vogelstein, 1983; Ariel et al., 1995; Wutz et al., 1997). For many years, DNA methylation has been regarded as long lasting and an epigenetic lock on transcription. Cases of highly dynamic regulation of DNA methylation were reported by the Gannon and Reid laboratories (Kangaspeska et al., 2008; Metivier et al., 2008), suggesting cyclical methylation/demethylation at promoters as a part of the transcription cycle at least at some promoters. They showed that the process involves DNA methyltransferases (DNMTs) as well as thymine-DNA-glycosylase (Tdg). The recently discovered active DNA-demethylation pathway involves ten-eleven translocation (Tet) enzymes (Kriaucionis and Heintz, 2009; Tahiliani et al., 2009; Cortellino et al., 2011). Tet enzymes catalyze conversion of 5mC to 5-hydroxymethylcytosine (5hmC) and can further oxidize 5hmC to 5-formylcytosine (5fC) and 5-carboxylcytosine (5caC). Tdg can excise the latter two derivatives through base-excision-repair process that will generate un-methylated cytosines (Cortellino et al., 2011; He et al., 2011; Ito et al., 2011) closing the methylation cycle. The breakthrough discovery that Tet enzymes can oxidize 5-methylcytosine has revolutionized the concepts in the epigenetic field in general and the DNA methylation field in particular.

### DNA-METHYLATION AND ITS READERS

The relationship between DNA methylation and transcriptional silencing has proven challenging to decipher. Essentially, two types of mechanisms have been put forward: in the first a methylated cytosine physically inhibits binding of transcriptional regulators. It was reported that the binding of transcription factors such as AP-2 (Comb and Goodman, 1990), c-Myc (Prendergast et al., 1991), NF-kB (Bednarik et al., 1991), E2F1 (Kovesdi et al., 1987), and CREB (Iguchi-Ariga and Schaffner, 1989), is affected by DNA methylation.

The prevalent model proposes that proteins bind directly to methylated DNA – the so-called readers – recruit co-repressor complexes (Klose and Bird, 2006) and trigger the formation of repressive chromatin. Methylated DNA readers fall into three main classes: (i) proteins containing a methyl-CpG-binding domain (MBD), (ii) Kaiso and Kaiso-like family of proteins including ZBTB4-33-38, characterized by the presence of BTB/POZ domain and several Kruppel-like C2H2 zinc fingers, and (iii) SRA (SET and RING finger associated) domain containing proteins, like UHRF1 and UHRF2 characterized by the SRA domain that recognizes methylated DNA. Recently, a DNA pull-down screen followed by Mass Spectrometry has uncovered additional 5mC and 5hmC readers that do not belong to any of the three families listed above (Spruijt et al., 2013), such as some homeobox and Rfx proteins.

### THE MBD FAMILY OF PROTEINS

**FIGURE 1:**
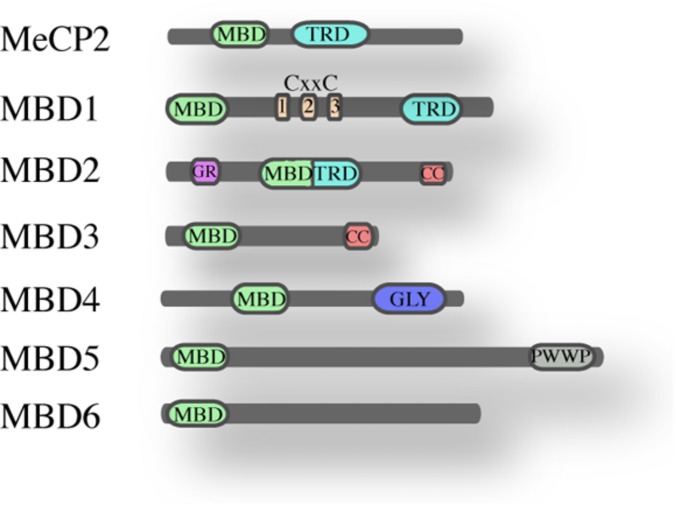
**The MBD family of proteins.** Schematic overview of the mammalian MBD family and their known domains (MBD, methyl-CpG-binding domain; TRD, transcriptional repression domain; CXXC, Zinc finger-Cys-x-x-Cys domain; G/R, arginine-rich; CC, coiled-coil; GLY, glycosylase; PWWP, Pro-Trp-Trp-Pro).

The founding member of the MBD family is MeCP2 (Lewis et al., 1992), a protein of 53 kDa containing an N-terminal MBD (Lewis et al., 1992; Nan et al., 1993) and a C-terminal transcription repression domain (TRD; Nan et al., 1998). MeCP2 is ubiquitously expressed and highly abundant in the brain. Mutations in the gene encoding MeCP2 cause the Rett syndrome (Amir et al., 1999) and other neurodevelopmental disorders (Moretti and Zoghbi, 2006). Crystal structure of the MBD domain, in complex with methylated DNA, showed that it binds symmetrical methylated CpGs *in vitro*. The MBD domain of MeCP2 – a domain of 70 amino acids – has been used in homology searches that led to the identification of six additional family members named MBD1 to MBD6 (**Figure 1**; Hendrich and Bird, 1998). Other studies revealed four additional proteins that contain an MBD-like domain namely SETDB1, SETDB2 (Schultz et al., 2002), BAZ2A and BAZ2B (Jones et al., 2000).

MBD1, MBD2, and MBD4 can bind methylated DNA *in vitro*, while MBD3, MBD5, and MBD6 do not appear to bind methylated DNA, at least not *in vitro* (Laget et al., 2010). A recent study on MBD5 and MBD6 revealed that their MBD domains interact with the human PR-DUB complex (Baymaz et al., 2014). MBD1, MBD2, MeCP2, and Kaiso appear not to be essential for development since their deletion is not embryonic lethal whereas MBD3 plays an essential role in embryonic development (Hendrich et al., 2001a; Martin Caballero et al., 2009).

MBD2 and MBD3 are close relatives and probably descend via gene duplication from an ancestral MBD2/MBD3, that is present in some metazoans as for instance *Caenorhabditis elegans* and *Drosophila* (Marhold et al., 2004). Outside the MBD domain, MBD2 and MBD3 share almost 80% homology; they both have an MBD and a coiled-coil domain (CC). Apart from this common domain, MBD2 contains an additional N-terminal glycine-arginine (GR) rich domain and a transcriptional repressor domain (TRD), whereas MBD3 has a C-terminal poly-glutamate region. Three isoforms have been described for MBD2 protein: the full length MBD2a, MBD2b lacking the N-terminal GR repeat and MBD2c that is a testis specific isoform lacking the C-terminus. Also MBD3 presents three isoforms: Mbd3b – the major isoform in embryonic stem cells, Mbd3a and a smaller isoform Mbd3c (Kaji et al., 2006). The crucial difference between MBD2 and MBD3 is that MBD3 does not bind methylated DNA, because it lacks four conserved amino acids in the MBD domain.

## MBD PROTEIN COMPLEXES: LOOKING FOR FUNCTIONS

Biochemical data provided strong evidence that methyl-CpG-binding domain proteins (MBPs) are an integral part of chromatin-remodeling complexes reported to mediate heterochromatin formation and transcriptional silencing. MeCP2 transiently interacts with the Sin3A and HDAC2 complexes (Nan et al., 1998), and MBD1 with SETDB1, SUV39H, and HP1 (Fujita et al., 2003; Sarraf and Stancheva, 2004). MBD2 and MBD3 are integral parts of the Mi-2/NuRD complex (Zhang et al., 1999; Le Guezennec et al., 2006). Mi-2/NuRD complex is containing ATP-driven chromatin remodeling and histone deacetylase activities. Protein interaction studies have demonstrated that MBD2 and MBD3 bind HDAC1/2 (Wade et al., 1999; Zhang et al., 1999), GATAD2A (p66α) and GATAD2B (p66β; Brackertz et al., 2002; Saito and Ishikawa, 2002; Gnanapragasam et al., 2011). Initially MBD2 and MBD3 were thought to be part of the same complex. It was postulated that Mi-2/NuRD containing both MBD2 and MBD3 is recruited to methylated DNA via MBD2 (Zhang et al., 1999). Knockout studies however showed that MBD2 and MBD3 have distinct functions because MBD3, but not MBD2, is embryonic lethal (Hendrich et al., 2001b). Indeed biochemical analyses showed that MBD3 and MBD2 are mutually exclusive within the Mi-2/NuRD complex (Le Guezennec et al., 2006). In addition to the already known Mi-2/NuRD components, DOC-1 was identified as a novel subunit of both MBD2-NuRD (Le Guezennec et al., 2006; Spruijt et al., 2010) and MBD3-NuRD (Spruijt et al., 2013).

## MBD PROTEINS IN TRANSCRIPTION REGULATION

The role of MBPs in transcription regulation has been widely studied. MBPs have often been associated with transcriptional repression because of their interaction with co-repressor complexes triggering heterochromatin formation. However, a unifying role and mechanism have yet to be established. Informative approaches to decipher the function of a protein are knock-down (KD), knock-out (KO), and overexpression studies. Surprisingly, knock-down as well as overexpression of MeCP2 have been reported to cause both transcription activation and repression in the hypothalamus, with 85% of target genes being activated by MeCP2 (Chahrour et al., 2008). Also the KD of MBD2 resulted in both transcriptional activation and repression. For instance it has been shown that in adult erythrocytes, MBD2 and MTA2 – a Mi-2/NuRD subunit – are enriched at the inactive ρ-globin gene when this gene is highly methylated and repressed. MBD2 KD resulted in re-expression of the ρ-globin gene (Kransdorf et al., 2006). Another direct target of MBD2-mediated repression is the Il4 gene, whose level of expression is increased in T cells derived from Mbd2-null mice (Hutchins et al., 2002). MBD2 was also reported to affect the Xist gene silencing in mouse: Mbd2 knock-down cells display lower level reactivation of *Xist* whereas silencing was rescued by re-expression of Mbd2 (Barr et al., 2007).

In a recent study, the link between MBD2 binding and expression changes of neighboring genes was assessed using chromatin immunoprecipitation (ChIP) experiments following MBD2 depletion (Günther et al., 2013). Only mild alterations in gene expression were observed. Moreover, after MBD2 depletion, gene expression changes revealed a roughly equal number of genes that were up or down-regulated. As mentioned above, the level of DNA methylation nor imprinting are affected in MBD2 null mice suggesting that it is not required for correct silencing of imprinted genes.

Taken together, the generally accepted model of MBD2 acting as a transcriptional repressor has not been unambiguously supported by experimental data and a unifying model of the molecular mechanism is missing.

## MBD2 AND MBD3: THE COMPLEX BALANCE OF PLURIPOTENCY/REPROGRAMMING

MBD2 has two isoforms: the full length MBD2a and a testis specific isoform MBD2c, lacking the C-term (Hendrich and Bird, 1998). The C-term domain of MBD2 is responsible for the interaction with p66α (GATAD2A) and therefore with the Mi2-NuRD complex (Brackertz et al., 2002; Gnanapragasam et al., 2011). Protein interaction studies have shown that MBD2c is unable to bind Mi-2/NuRD components such as Mi-2β, HDAC1 and HDAC2 (Baubec et al., 2013). A recent study showed that in human pluripotent stem cells the two isoforms sustain different pathways: MBD2a promotes differentiation and its overexpression disrupts pluripotency while MBD2c facilitates reprogramming of fibroblasts (Lu et al., 2014). Moreover this study shows that the 3′ UTR of MBD2a is a direct target of the miR-302, previously reported to promote reprogramming (Subramanyam et al., 2011) via up-regulation of NANOG expression and suppression of MBD2 (Lee et al., 2013). These recent findings shed new light on the role of MBD2 in regulating the commitment toward either reprogramming or differentiation, involving the microRNA and splicing factors. Further analysis will be needed to dissect the transcriptional and epigenetic changes in response to deletion of either isoforms, in order to identify specific targets and downstream effectors that might be mediating either lineage commitment or retention of pluripotency.

The role of MBD3 in lineage commitment and pluripotency has recently been at the focus of attention (Yildirim et al., 2011; Reynolds et al., 2012; Whyte et al., 2012). The importance of MBD3 in regulating escape from pluripotency and lineage commitment has been documented in several studies. Early reports described Mbd3 as essential for lineage commitment, since Mbd3 depleted mouse embryonic stem cell failed to differentiate and aberrantly self-renewed independently of leukemia inhibitory factor (LIF), one of the essential factors needed to keep undifferentiated stem cells in culture (Kaji et al., 2006). Recently two independent studies showed that Mbd3 constitutes a gate to full reprogramming and that its depletion together with transduction of the four Yamanaka factors (Oct4, Sox2, Klf4, Myc, all together OSKM) enhances the reprogramming efficiency, consistent with the role of Mbd3 as suppressor of reprogramming (Luo et al., 2013; Rais et al., 2013). However, other findings suggest an opposite role for Mbd3 in facilitating induction of pluripotency (Dos Santos et al., 2014). The authors themselves underline that the differences with respect to previous studies might be due to a different cell system and reprogramming conditions, and that their findings may be context specific. However, reproducibility of previous results seemed to be somewhat challenging, raising more questions than providing answers.

## MBPs IN THE ORGANIZATION OF CHROMATIN STRUCTURE

Several studies have pointed to a role of MBD proteins in the organization of chromatin structure and shaping the local and global epigenome landscape, for instance formation or maintenance of heterochromatic, repressive chromatin (Clouaire and Stancheva, 2008; Fournier et al., 2012), given the presence of an ATP-driven remodeler subunit within the Mi-2/NuRD complex (Wade et al., 1999; Zhang et al., 1999).

Little is known about the impact of MBD2 on chromatin organization *in vivo*. It has been observed that mouse myoblasts undergoing myogenic differentiation form aggregates of pericentric heterochromatin (chromatin surrounding the centromere) that display increased levels of MeCP2, MBD2, and DNA methylation (Brero et al., 2005; Luo et al., 2009). Overexpression of MBD2 or for that matter MeCP2 induced condensation of densely stained heterochromatin within the nucleus. Interestingly also in the absence of MeCP2 (skeletal muscle tissue derived from MeCP2 knock-out mice), MBD2 overexpression triggered the formation of densely stained repressive chromatin, suggesting some degree of functional redundancy or overlap. The molecular mechanisms behind this aggregation of pericentric chromatin remain unclear. A recent study shows that in human cells ectopic expression of MBD2, but not MBD3, induces heterochromatin compaction (Günther et al., 2013). To resolve the putative role of MBD2 in the formation of higher order chromatin structure it will be important to combine genome-wide localization studies with chromosome conformation capture approaches. A first study along these lines (Shimbo et al., 2013) indeed suggests that MBD3 binding to enhancers results in their closer proximity to promoters and gene bodies because of protein mediated looping.

## GENOME-WIDE MBD2 AND MBD3 BINDING SITE ANALYSES

Chromatin immunoprecipitation followed by next generation sequencing, (ChIP-seq) is widely used to determine the binding sites of a protein or transcription factor in a genome-wide manner. A substantial amount of literature describes the characterization of MBD2 and MBD3 binding to chromatin at specific loci but only recent studies provided genome-wide maps. ChIP experiments on chromatin regulators, such as Mi-2/NuRD complex, turned out to be quite challenging, presumably because of a combination of factors: low abundancy of the proteins, the transient nature of the chromatin association, short residence time on chromatin and the quality of the antibodies.

**FIGURE 2:**
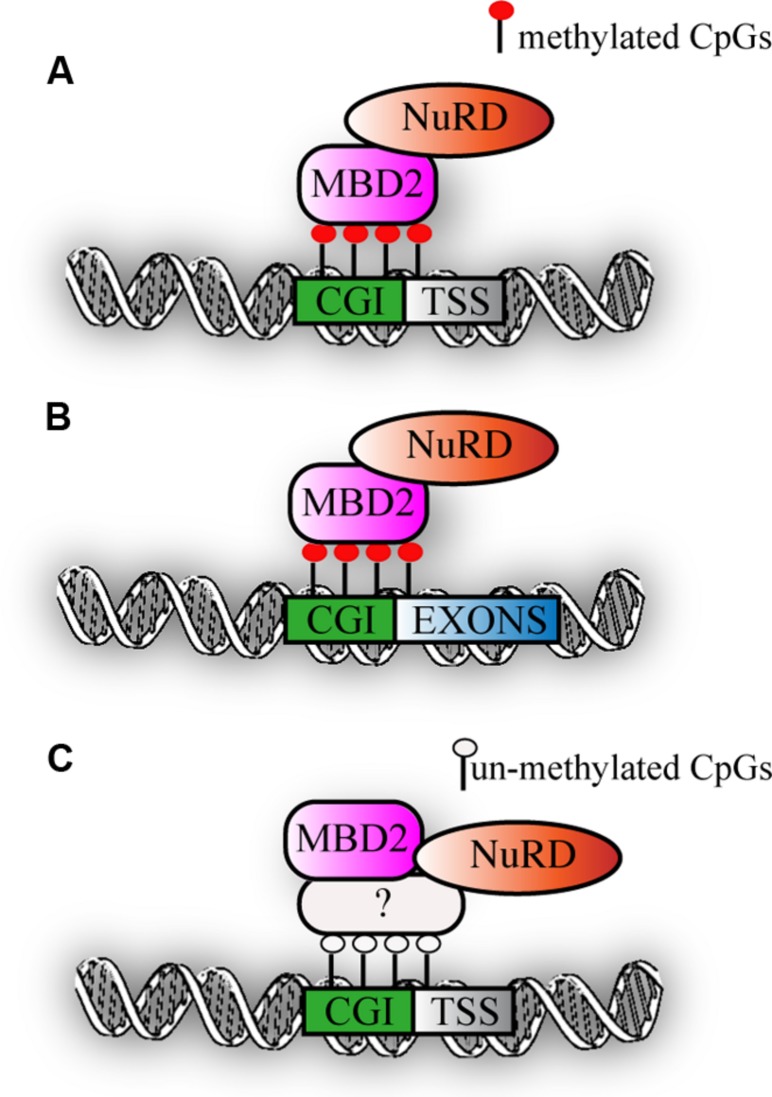
**MBD2 binding to chromatin. (A)** Schematic representation of MBD2 binding to highly methylated CGI promoters **(B)** MBD2 binding to highly methylated CGI exons. **(C)** Subset of MBD2 binds to un-methylated active promoters, this binding depends on the interaction with Mi-2/NuRD complex that might be recruited to chromatin via other, not yet identified factors.

Recent genome-wide mapping of MBPs has clearly shown that MBD2 binds to highly methylated, CpG dense regions *in vivo* (Baubec et al., 2013). Interestingly, motif analysis did not reveal any specific DNA sequence motif within the MBD2 binding sites and their flanking regions, but highlighted primarily CG richness of the binding sites as the critical factor for binding. In line with this, an intact methyl binding domain is essential to target MBD2 to densely methylated loci. DNA methylation is a prerequisite since binding of MBD2 is lost in cells with triple knock-out for Dnmt1-3a/b, that lack both methylation and hydroxymethylation. MBD2 primarly binds at highly methylated promoters (Günther et al., 2013; Menafra et al., 2014) and secondly exons (**Figures 2A,B**). Surprisingly, in mouse embryonic stem cells, a correlation between genome-wide MBD2 binding and known components of the Mi-2/NuRD complex such as Mi-2β and Hdac2 was not observed. It should be noted however that the profiles for the two subunits were not performed in parallel (Whyte et al., 2012). Interestingly, a subset of MBD2 binding sites have been uncovered that did not have DNA methylation. The presence of MBD2 at these loci was suggested to be due to its association with the Mi-2/NuRD complex that was recruited to chromatin via other, not yet identified factors (**Figure 2C**). Indeed, MBD2 binding at such loci was lost when the testis specific MBD2 isoform was expressed that lacks the C-terminus and does not immunoprecipitate any of the known Mi-2/NuRD components. Surprisingly, Baubec et al. also found that MBD2 is recruited to a subset of un-methylated loci that displayed epigenetic marks pointing at active regulatory regions. These data were confirmed in another independent study in which a tagged MBD2 was expressed in human MCF-7 cells. A small fraction of MBD2 binding sites was observed at promoters of genes displaying active histone marks and low gene expression (Menafra et al., 2014). Interestingly, the study from Günther et al. revealed a dichotomy within MBD2 binding to promoters of silent genes and at the same time to exons of actively transcribed genes, suggesting that in the latter case, MBD2 might play a role in splicing. It remains to be elucidated what the effects of MBD2 binding are on transcription and chromatin organization.

Identification of genome-wide binding sites and functional analyses of MBD3 have been performed in mouse (Yildirim et al., 2011; Baubec et al., 2013) and human cells (Günther et al., 2013; Shimbo et al., 2013) reaching different conclusions. Yildirim et al. reported that MBD3 binds just downstream of the transcription start site (TSS) of CpG-rich promoters marked by hydroxymethylation. Baubec et al. found, however, that MBD3 binds to active regulatory regions (enhancers) independent of CpG density and independent of their methylation or hydroxymethylation status, since binding was maintained in Dnmt1/3a/3b triple knock-out embryonic stem cells. These data question the validity of the binding of MBD3 to hydroxymethylated DNA, which was further underpinned by DNA pull-downs followed by MS analysis (Spruijt et al., 2013), which did reveal hydroxymethylation specific readers but in which MBD3 was not detected.

Independent studies in human cells (Günther et al., 2013; Shimbo et al., 2013) both suggested that MBD3 is localized at CpG rich promoters of active genes, but while Günther et al. (2013) showed that it mainly binds promoters, Shimbo and coworkers suggested a more complex regulation of both active and silent genes. They showed that a fraction of MBD3 binds at enhancers that are in physical proximity to promoters and gene bodies in three-dimensional space and hence are picked up in the assay. Since these data have been generated by different groups, using different antibodies, cell systems, and sequencing analysis pipelines (see **Table 1**, for a summary of the different studies), re-analysis of the data with a common approach may resolve some of the major and minor discrepancies. Interestingly, the genome-wide binding map of MBD3 correlates to some extend with that of another Mi-2/NuRD component, Mi-2β, implying partial co-recruitment of these two subunits on chromatin. Further studies will be needed to assess whether this interaction is necessary for Mi-2/NuRD to bind the chromatin and whether MBD3 depletion would result in loss of Mi-2β binding or *vice versa*. Similarly as reported for MBD2, depletion of MBD3 in human (Günther et al., 2013) and mouse cells (Yildirim et al., 2011) resulted in alteration of gene expression for only a small number of genes and in both cases, the transcriptional changes are rather mild.

**Table 1:** Summary of studies characterizing MBD3 binding genome-wide: technical details of the experiments together with findings and algorithm used for the analysis are listed.

Study	Organism	Cell type	Insert	Antibody	Localization	Chromatin features	Number of peaks/enriched regions	Algorithm used for analysis
Yildirim et al. (2011)	Mouse	ES	NA (endogenous)	Mix of (ab3755) and Bethyl (A302-528A)	Downstream TSS	Hydroxy-methyl, Tet1	NA	NA
Baubec et al. (2013)	Mouse	ES	MBD3 biotinylated	Streptavidin beads	Unmethylated regions	DNaseI, H3K4me1, and H3K27ac	NA	Enrichment over input in a 1 kb sliding window
Günther et al. (2013)	Human	HeLa	MBD3-V5	V5 agarose (Sigma Aldrich A7345)	CpG rich, unmethylated promoters	H3K4me2/3; H3K9ac; DNAse1; FAIRE	490	Peak-ranger
Shimbo et al. (2013)	Human	MCF-7	NA (endogenous)	Ab91458	Promoters, gene bodies and enhancers of active genes	Five patterns:mainly H3K27ac (not TSS) and H3K4me3 TSS	35165	SICER
Shimbo et al. (2013)	Human	MDA-231	NA (endogenous)	Ab91458	Promoters, gene bodies and enhancers of active genes	Five patterns:mainly H3K27ac (not TSS) and H3K4me3 TSS	23880	SICER

**FIGURE 3:**
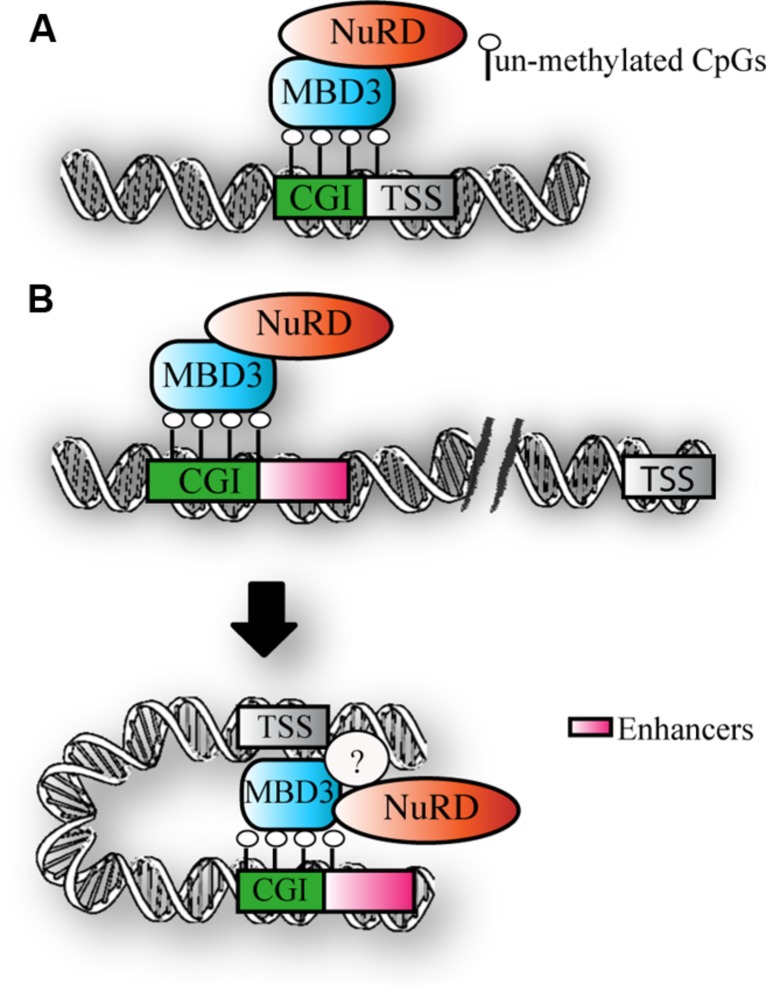
**MBD3 binding to chromatin. (A)** Schematic representation of MBD3 binding to unmethylated CGI promoters **(B)** MBD3 binding to unmethylated enhancers, that are in physical proximity to promoters in three-dimensional space. The question mark indicates the possible presence of other subunits involved in this association.

Taken together, the above findings suggest that MBD3 binds CpG-rich active promoters and enhancers that are not DNA methylated (**Figure 3**). The downstream functional consequence of MBD3 binding has still to be elucidated.

## CONCLUDING REMARKS

Since its discovery in 1975, DNA methylation has been one of the best studied epigenetic marks and the readers of DNA methylation that translate the signal of methylated DNA into a function or activity have obtained a lot of attention. MBD2 and MBD3 are two very similar proteins, which are interchangeable structural parts of the Mi-2/NuRD complex and share many common subunits. Nevertheless, they appear to perform distinct functions and to differ completely in their ability to bind methylated DNA. The emerging picture is that MBD2 binds to CpG-rich, densely methylated DNA *in vivo*, with an apparent but not understood preference for promoter regions. A minor fraction of MBD2 is present at promoters that bear active epigenetic marks and are not methylated. This is the only fraction of binding sites depending on the interaction with Mi-2/NuRD, suggesting that MBD2 might also have a function independent of Mi-2/NuRD. Recruitment of Mi-2/NuRD might also depend on subunits other than MBD2 such as GATAD2A-B or MTA1-2 that is reported to directly bind the histone H3 tail *in vitro* (Robertson and Jones, 2000; Wu et al., 2013). Supporting *in vivo* data is currently not available. Therefore additional biochemical studies followed by *in vivo* evidence, for example by ChIP-seq, should be performed to assess whether MBD2 exists in distinct complexes and can bind to unmethylated regions independent of Mi-2/NuRD. MBD3 binds CpG-rich, unmethylated active promoters and enhancers. One of the burning questions is how MBD3/NuRD is recruited to its genomic binding sites. Does MBD3 play a role, are other subunits important or is the association based on transient protein-mediated interactions? What seems clear is that MBD3 plays an important role in lineage commitment.

The biological functions of MBD2 and MBD3 remain an open question; whether and how they modulate transcriptional activity and chromatin structure. The recent coupling of chromosome conformation capture technologies with deep-sequencing might provide an interesting angle to look for possible participation of MBD2 in long range interactions, a model proposed for MBD3 but not yet addressed in a genome-wide fashion. Even after decades of research, DNA methylation readers are still hiding their true nature, raising puzzling but at the same time intriguing questions for further research.

## Conflict of Interest Statement

The authors declare that the research was conducted in the absence of any commercial or financial relationships that could be construed as a potential conflict of interest.
